# Cross-Modality Alignment of Spatial Transcriptomics, Multiplexed Imaging, and Histology with PHARAOH

**DOI:** 10.21203/rs.3.rs-9954002/v1

**Published:** 2026-06-19

**Authors:** Sicong Yao, Zehua Jing, Shunzhou Jiang, Wei Li, Yoshiaki Yamamoto, Bernhard Dumoulin, Nadja Sachs, Lars Maegdefessal, Katalin Susztak, Linghua Wang, Humam Kadara, Mingyao Li

**Affiliations:** 1. Statistical Center for Single-Cell and Spatial Genomics, Department of Biostatistics, Epidemiology and Informatics, University of Pennsylvania Perelman School of Medicine, Philadelphia, PA, USA.; 2. Renal, Electrolyte, and Hypertension Division, Department of Medicine, University of Pennsylvania Perelman School of Medicine, Philadelphia, PA, USA.; 3. Penn/CHOP Kidney Innovation Center, University of Pennsylvania Perelman School of Medicine, Philadelphia, PA, USA.; 4. Department for Vascular and Endovascular Surgery, TUM Klinikum Rechts der Isar, Technical University of Munich, Munich, Germany; 5. German Center for Cardiovascular Research (DZHK), Berlin, Germany; partner site Munich Heart Alliance, Germany.; 6. Institute of Molecular Vascular Medicine, TUM Klinikum Rechts der Isar, Technical University Munich, Munich, Germany.; 7. Department of Medicine, Karolinska Institutet, Stockholm, Sweden.; 8. Department of Genomic Medicine, UT MD Anderson Cancer Center, Houston, TX, USA.; 9. The University of Texas MD Anderson Cancer Center UTHealth Houston Graduate School of Biomedical Sciences, Houston, TX, USA.; 10. The James P. Allison Institute, The University of Texas MD Anderson Cancer Center, Houston, TX, USA.; 11. Institute for Data Science in Oncology, UT MD Anderson Cancer Center, Houston, TX, USA; 12. Department of Translational Molecular Pathology, UT MD Anderson Cancer Center, Houston, TX, USA.; 13. Department of Head and Neck Surgery, UT MD Anderson Cancer Center, Houston, TX, USA.; 14. Department of Pathology and Laboratory Medicine, University of Pennsylvania Perelman School of Medicine, Philadelphia, PA, USA.

## Abstract

Accurate cross-modality alignment between spatial transcriptomics (ST), multiplexed imaging, and histology is essential for spatially contextualized molecular analysis. In imaging-based ST platforms, molecular measurements are defined in the coordinate system of nuclear-stained images rather than in histological space, requiring precise registration for accurate integration with tissue morphology. Existing approaches either rely on labor-intensive manual landmark placement or lack the precision required for cellular-resolution alignment. Here we present PHARAOH, a hierarchical framework for cross-modality alignment of ST, multiplexed imaging, and histology. By combining global structural alignment with automated nuclear landmark anchoring, PHARAOH enables accurate coordinate transfer between molecular imaging and histological space with minimal user input. Across diverse tissue types, imaging platforms, and staining conditions, PHARAOH consistently achieved fast, robust, and spatially precise alignment, including across adjacent tissue sections. The framework further generalizes to multiplexed immunofluorescence imaging, providing a unified and scalable strategy for integrating spatial molecular measurements with histological architecture.

## Introduction

Spatial transcriptomics (ST) technologies enable the measurement of gene expression within intact tissue architecture, preserving spatial context that is lost in dissociated single-cell sequencing^1,2^. Early sequencing-based ST platforms generate spatially resolved transcriptomic maps at spot-level resolution, offering valuable insights into tissue organization but remaining limited in spatial granularity and direct morphological interpretability^3,4^. Recent advances in imaging-based ST platforms directly visualize labeled transcripts in situ at near single-cell or even subcellular resolution^5–7^. By resolving transcript localization within individual cells and microanatomical structures, imaging-based ST tightens the coupling between molecular measurements and tissue architecture, enabling more precise interrogation of cellular microenvironments and spatial heterogeneity^8,9^.

Complementing these molecular measurements, hematoxylin and eosin (H&E) staining remains the gold standard for visualizing tissue morphology in histopathology, providing detailed delineation of cellular boundaries and structural organization^10–12^. Such morphological information is critical for contextualizing spatial gene expression patterns^13,14^. In practice, many ST platforms include matched H&E-stained histological images, enabling integrated molecular–morphological analysis and multimodal learning^3,6,15,16^. For imaging-based ST technologies, however, molecular measurements and histological images are frequently generated through separate acquisition pipelines, thereby residing in independent coordinate systems^6,17^. As spatial resolution approaches cellular scale, accurate spatial correspondence between modalities becomes increasingly critical^18^. Even small misalignments between H&E images and ST data can disrupt the mapping of transcripts to their correct histological context, particularly near cellular boundaries, thereby confounding downstream spatial analyses. Without precise alignment, cell-level relationships cannot be reliably established, rendering accurate cross-modality registration essential for meaningful biological interpretation.

Imaging-based ST platforms typically incorporate a nuclear stain channel, such as 4’,6-diamidino-2-phenylindole (DAPI), to support cell segmentation and spatial localization^6^. Because hematoxylin contrast in H&E reflects nuclear chromatin architecture and DAPI fluorescence directly labels nuclear DNA, the two modalities encode highly concordant nuclear spatial patterns^19–22^. These conserved nuclear geometries provide a strong structural basis for cross-modality registration, making DAPI-to-H&E alignment a common strategy for integrating molecular imaging data with histological context. Existing registration approaches generally rely either on global feature-based frameworks, such as VALIS^17^, or on manually guided alignment using either platform-specific tools (e.g., Xenium Explorer) or general-purpose image analysis software (e.g., Fiji/ImageJ). While these strategies can achieve reasonable correspondence under favorable conditions, high-resolution imaging-based ST demands cell-level precision and robustness against variability in staining quality. Global feature-based approaches may not achieve the pixel-level geometric precision required for accurate cell- or transcript-level coordinate transfer, and manual landmark selection is labor-intensive, operator-dependent, and prone to selection bias, limiting reproducibility and scalability. In addition, whole-slide registration methods may require substantial computational resources or extensive parameter tuning when applied to ultra-high-resolution imaging-based ST datasets, limiting their practicality for standardized and scalable analysis workflows. Consequently, there remains a need for an automated, scalable, and structurally informed registration framework that generalizes across imaging-based ST platforms.

To address these challenges, we present PHARAOH (**P**yramid-based **H**ybrid **A**nchor-driven **R**obust **A**lignment for spatial **O**mics and **H**istology), a robust landmark-driven hierarchical registration framework for ST, multiplexed imaging, and histology. By combining global structural initialization with automated nuclear landmark anchoring, the framework achieves pixel-level geometric precision while requiring only minimal user input. Across diverse imaging-based ST platforms and tissue types, including cases with suboptimal staining or imaging artifacts, PHARAOH consistently enables accurate cell- and transcript-level coordinate transfer between molecular imaging and histological sections. The method further enables alignment between adjacent tissue sections through histology-anchored coordinate transfer, facilitating reliable cross-section spatial mapping and ST-ST integration. Beyond ST, the framework generalizes to multiplexed immunofluorescence, enabling consistent coordinate transfer across diverse protein channels and supporting integrated cross-modality spatial analysis.

## Results

### Overview of the PHARAOH framework

PHARAOH establishes precise spatial correspondence between nuclear-resolved fluorescence imaging and histological sections, a critical yet technically challenging step in imaging-based ST workflows. In platforms such as 10x Genomics Xenium, accurate cross-modality registration is essential for transferring molecular measurements into histological space.

**Fig. 1 |:**
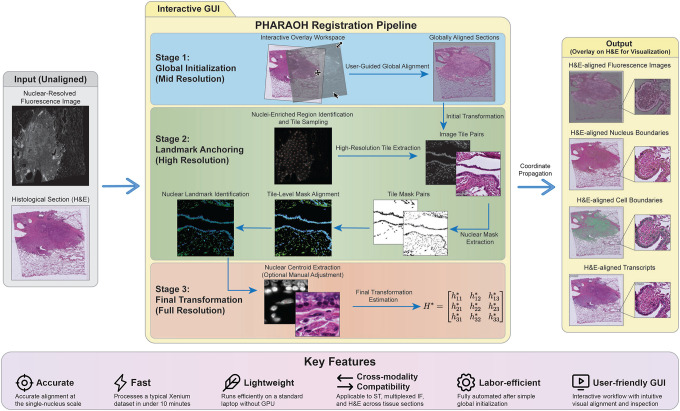
Overview of PHARAOH pipeline. PHARAOH aligns nuclear-resolved fluorescence images (e.g., DAPI) with H&E sections through a three-stage pipeline. First, an interactive GUI enables user-guided global initialization at mid-resolution to correct large-scale spatial discrepancies. Second, nuclei-enriched regions are automatically identified and sampled into tiles, where nuclear masks are extracted and aligned to establish local correspondences and refine landmark matching at high resolution. Third, nuclear centroids are used to estimate a final full-resolution transformation. The resulting mapping enables coordinate propagation from fluorescence to histology space, producing H&E-aligned fluorescence images, cell and nucleus boundaries, and transcript coordinates.

Histological interpretation itself is inherently hierarchical, where global tissue architecture is first assessed before focusing on local cellular features^23^. Motivated by this principle, PHARAOH addresses the registration challenge through a hierarchical multi-scale framework that enables accurate coordinate mapping from molecular imaging into the histological reference frame while requiring minimal human input (Fig. 1).

The framework integrates global alignment and local refinement within a multi-resolution architecture. At an intermediate resolution, a lightweight user-guided initialization establishes global alignment between image modalities, correcting large-scale spatial discrepancies such as translation, rotation, scaling, and perspective differences between modalities. Subsequently, nuclei-enriched regions are automatically identified and sampled as high-resolution tiles for local refinement. Within these regions, nuclear structures are extracted and used to establish local correspondences, enabling precise alignment at the cellular scale

Once the cross-modality mapping is established, spatial coordinates from molecular imaging, including cell centroids and transcript locations, can be reliably propagated into the histological reference frame. This enables direct interpretation of molecular measurements within tissue morphology and supports downstream analyses that depend on accurate spatial localization.

By combining coarse global alignment with fine-scale nuclear anchoring, PHARAOH achieves high spatial accuracy while maintaining computational efficiency across large tissue sections. The multi-resolution design constrains local refinement within globally consistent regions, reducing error propagation and improving robustness to staining variability while substantially reducing the computational burden associated with exhaustive whole-slide high-resolution registration. As a result, the framework remains computationally lightweight and can be executed on standard desktop-scale computing hardware without requiring specialized GPU acceleration.

To facilitate practical use, PHARAOH is accompanied by an interactive graphical user interface (GUI) that organizes the registration process into a guided, stage-based workflow. This design reduces manual effort and enhances usability and reproducibility across diverse datasets and experimental conditions.

### High-accuracy DAPI–H&E registration in imaging-based ST

Imaging-based ST platforms such as Xenium acquire molecular imaging data together with nuclear fluorescence channels that provide structural cues for spatial localization. Because histological sections are generated through independent acquisition pipelines, accurate spatial transfer requires robust cross-modality registration between fluorescence nuclear images and histological sections. To evaluate the performance of PHARAOH, we applied the framework to multiple Xenium datasets generated by the 10x Genomics platform, including both publicly available benchmark datasets and real-world samples obtained through collaborative studies.

**Fig. 2 |:**
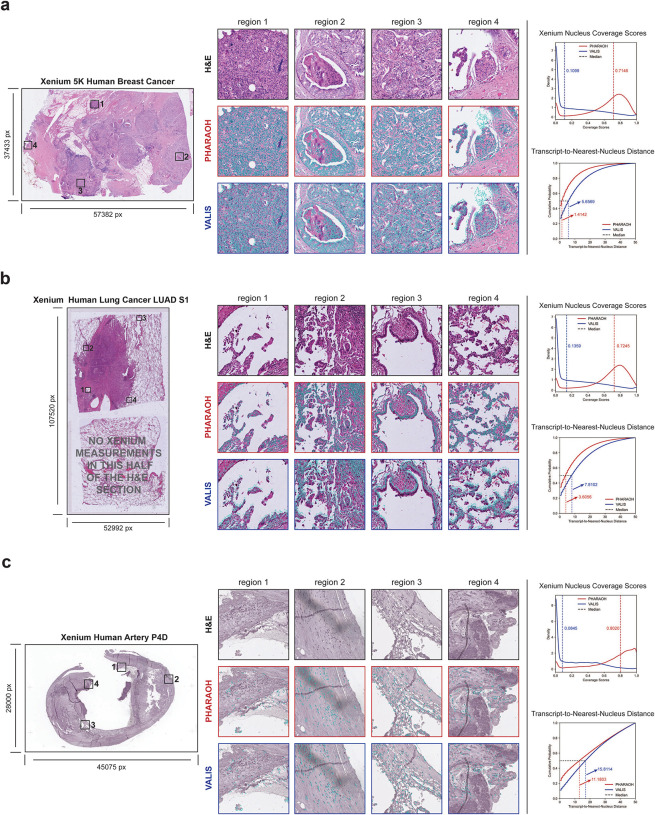
Evaluation of PHARAOH alignment accuracy across Xenium dataset. **a–c**, Representative regions from human breast cancer, LUAD, and carotid artery datasets. For each dataset, H&E images (top row) are compared with overlays of Xenium-measured nucleus boundaries mapped to H&E space using PHARAOH (middle row) or VALIS (bottom row) across multiple regions of interest (regions 1–4). PHARAOH shows improved correspondence between nuclear signals and hematoxylin-stained nuclei, particularly in complex or low-quality regions. Right, quantitative evaluation: nucleus coverage score distributions (top, computed based on H&E nucleus segmentation; see Methods) indicate substantially higher coverage of transcripts within nuclear regions for PHARAOH compared to VALIS. Across datasets, PHARAOH increased coverage scores with median differences of approximately 0.48–0.53, with higher coverage observed in 85–93% of regions. This corresponds to a strong paired effect size (matched-pairs rank biserial correlation, *r*_*rb*_ = 0.71–0.85), indicating consistent improvements across regions. Transcript-to-nearest-nucleus distance distributions (bottom) show reduced distances for PHARAOH compared to VALIS. Within the shared 0–50 pixel comparison range, PHARAOH decreased median distances by approximately 4.2–4.6 pixels across datasets. The distributions were consistently shifted toward smaller values (KS statistic = 0.15–0.20, P < 0.05), and sampled pairwise comparisons indicated that PHARAOH distances were smaller than VALIS in 57–63% of cases (common-language effect size, CLES = 0.57–0.63), consistent with improved cellular-level spatial correspondence.

Across diverse Xenium datasets spanning multiple tissue types, including human breast cancer, lung adenocarcinoma (LUAD), colorectal cancer, and liver cancer, as well as mouse brain and colon tumor samples, PHARAOH consistently established accurate spatial correspondence between DAPI nuclear images and matched H&E sections at the cellular scale. Representative regions from breast cancer, LUAD, and artery datasets are shown in Fig. 2a–c. Additional comparisons across all evaluated samples are provided in **Supplementary Figs. 1–3**. Visual inspection revealed strong concordance between Xenium-measured nucleus boundaries and hematoxylin-stained nuclear structures, demonstrating accurate nuclei-level alignment between fluorescence and histological images.

We next compared PHARAOH with VALIS^17^, a widely used whole-slide image registration framework based on generic feature matching. While VALIS provides modality-agnostic alignment, it does not explicitly leverage nucleus-to-nucleus correspondence, which is especially informative for DAPI–H&E registration. As a result, VALIS often exhibited local misalignments between DAPI nuclei and hematoxylin-stained nuclei, whereas PHARAOH achieved tighter nuclei-level correspondence with reduced spatial discrepancies (Fig. 2). In a subset of datasets, VALIS failed to produce a usable alignment or generated severely distorted results under the standardized computational setting used throughout this study (Methods) due to insufficient or unstable feature correspondence, whereas PHARAOH remained stable and produced accurate registrations; representative examples are shown in **Supplementary Fig. 4.**

This behavior was further examined in challenging datasets with low signal-to-noise DAPI images, such as the carotid artery dataset obtained from a patient with atherosclerosis^24^, which exhibited weak nuclear contrast and fragmented staining. Under these conditions, fully manual registration becomes highly difficult and impractical, as stable corresponding landmarks are hard to identify reliably across modalities. As a result, registration strategies that reduce the need for manual landmark identification are essential for achieving scalable and reproducible alignment. However, in such cases, feature-based matching methods such as VALIS can produce sparse or unstable correspondences, potentially affecting alignment reliability. By contrast, the nuclei-driven strategy of PHARAOH remained robust, as nuclear structures still provide informative geometric anchors even under degraded imaging conditions.

To quantitatively complement visual inspection, we measured nucleus coverage and transcript-to-nearest-nucleus distance (Fig. 2, **right**, Methods). These metrics provide indirect assessments of spatial consistency rather than definitive measures of alignment correctness, as ground-truth correspondence between modalities is not available. PHARAOH showed higher coverage of transcripts within nuclear regions and reduced distances between transcripts and their nearest nuclei compared to VALIS, consistent with improved cellular-level correspondence. These trends were particularly evident in tissue regions with complex morphology or reduced image quality.

Despite incorporating a user-guided initialization step, PHARAOH remains computationally efficient across diverse datasets. To evaluate its usability and robustness, three users with no prior experience using PHARAOH and one expert user (S.Y.) independently executed the full workflow on 16 Xenium datasets across heterogeneous computing environments (Methods, **Supplementary Methods**). To establish a baseline for fully manual registration, a separate independent user—not involved in the PHARAOH study—performed manual alignment using Xenium Explorer.

**Fig. 3 |:**
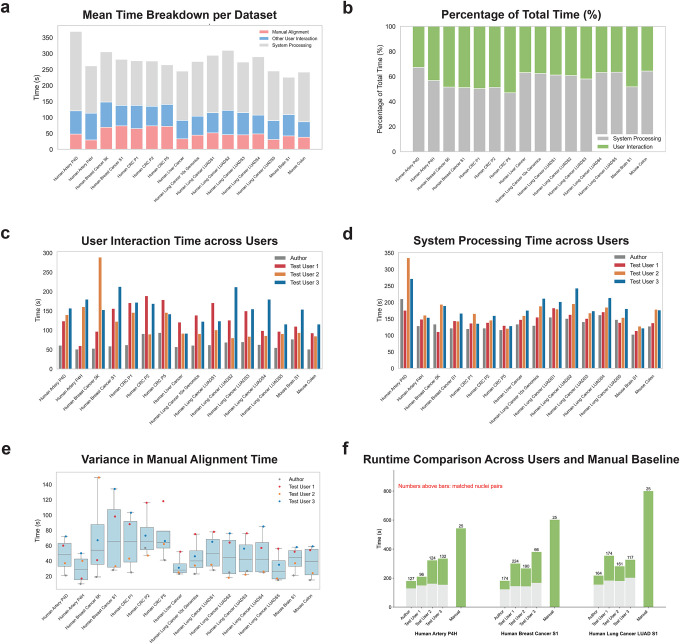
PHARAOH requires minimal user effort and shows consistent performance across independent users. **a**, Runtime breakdown across datasets, showing manual alignment, other user interaction, and system processing. System processing constitutes the dominant component of total runtime. **b**, Relative contributions of system processing and user interaction. System processing accounts for the majority of runtime across all datasets. **c**, User interaction time across multiple users. Interaction time is comparable across users, indicating low user-dependent variability. **d**, System processing time across users. System runtime is consistent across users for the same dataset, with variation driven primarily by dataset characteristics. **e**, Distribution of manual alignment time across datasets and users. Manual alignment times exhibit limited variability across users. **f**, Comparison between PHARAOH and fully manual registration using Xenium Explorer on a subset of datasets. Bars show per-user runtime for PHARAOH (stacked system and user components) and manual alignment, demonstrating substantially reduced user effort with PHARAOH. All timings exclude prolonged user inactivity (Methods, **Supplementary Methods**).

As shown in Fig. 3, PHARAOH maintained high efficiency across all datasets (Fig. 3a). Runtime analysis revealed that automated system processing dominates the workflow, while user interaction accounts for only a small fraction (Fig. 3b). The interactive component was highly stable, showing low variability across users, with consistent manual alignment time and system processing time across different hardware configurations and operating systems (Fig. 3c–e), indicating robustness to user-dependent and environment-dependent variation. Consequently, PHARAOH substantially reduced manual effort compared to fully manual alignment (Fig. 3f).

Collectively, these results demonstrate that PHARAOH is an accurate and robust framework for DAPI–H&E registration across diverse Xenium datasets. It consistently achieves nuclei-level correspondence under both standard and challenging imaging conditions with minimal manual intervention. The user-guided initialization step requires minimal training and enables reliable use by new users. By integrating nuclear structural information into an efficient and lightweight workflow, PHARAOH provides a practical and scalable solution for cross-modality spatial alignment in imaging-based.

### Scalable and robust registration for large-scale, spatially fragmented CosMx data

To further evaluate scalability and practical applicability, we applied PHARAOH to a CosMx spatial transcriptomics dataset from human kidney tissue^25^. Unlike whole-slide imaging platforms such as Xenium, CosMx acquires data as discrete fields of view (FOVs) that do not inherently form a continuous whole-slide representation^7^. As a result, the stitched DAPI image consists of spatially fragmented and partially disconnected regions, while molecular measurements are restricted to selected FOVs, posing a fundamental challenge for establishing reliable global correspondence between fluorescence and histological images.

**Fig. 4 |:**
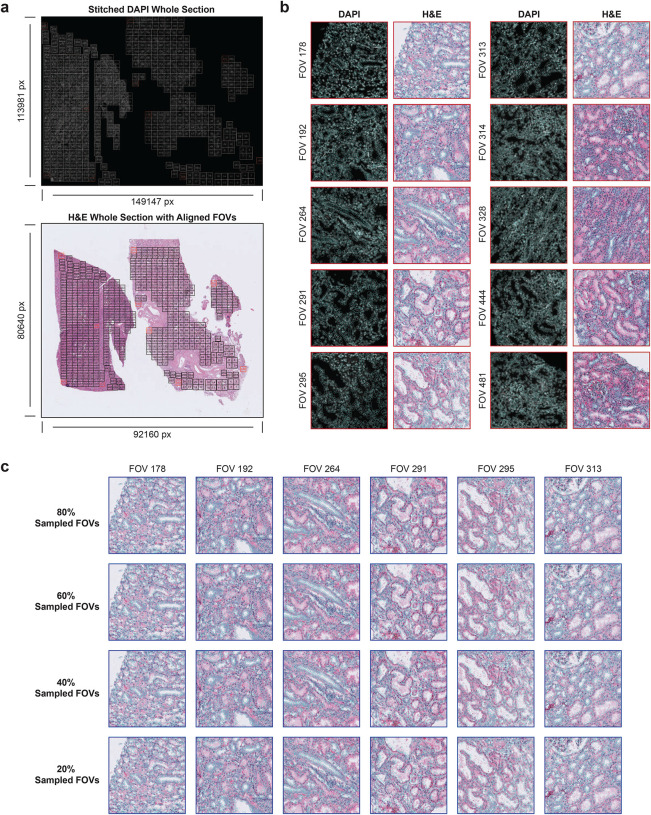
PHARAOH alignment of CosMx data under full and subsampled FOV stitching. **a,** Stitched DAPI whole-section image and corresponding H&E section with aligned FOVs. Selected FOVs of interest are highlighted in red, while remaining FOVs are shown with black or white outlines. **b**, Representative FOVs of interest from the fully stitched dataset (100% FOVs), showing DAPI images with CosMx-measured cell boundaries (left) and H&E images overlaid with PHARAOH-aligned cell boundaries (right), demonstrating accurate cross-modality mapping at cellular resolution. **c**, Robustness analysis under different FOV sampling proportions (80%, 60%, 40%, and 20%) used for stitching and alignment. Representative FOVs of interest show consistent alignment results across sampling levels, indicating that PHARAOH remains stable even with substantial subsampling of input FOVs.

The dataset comprised over 480 FOVs, each with a resolution of 4256 × 4256 pixels, which were computationally stitched into a composite DAPI image exceeding 149,000 × 113,000 pixels (Fig. 4a). The corresponding H&E whole-slide image reached 92,160 × 80,640 pixels. This scale, combined with fragmented spatial coverage, presents substantial challenges for conventional registration frameworks. Indeed, under the same standardized computational setting used throughout this study, VALIS failed to process this dataset due to excessive memory requirements associated with full-resolution image loading and global feature matching on standard desktop-scale hardware. In contrast, PHARAOH efficiently handled the dataset without excessive memory or CPU usage by leveraging pyramid-based image reading and localized tile sampling, enabling scalable registration across ultra-large and spatially fragmented tissue sections.

Manual alignment using general-purpose visualization tools such as Fiji or ImageJ is particularly challenging for CosMx data. The sparse and discontinuous spatial structure makes it difficult to identify corresponding landmarks, often requiring subjective selection of isolated anchor points. This process is time-consuming, difficult to reproduce, and prone to inconsistency. By contrast, PHARAOH first establishes global alignment at intermediate resolution, followed by automated tile-based refinement using nuclei-driven anchors, substantially reducing manual effort while improving robustness and consistency.

To evaluate alignment accuracy across the entire tissue section, we selected ten representative FOVs spanning the full specimen (Fig. 4b). Notably, the dataset contained three spatially separated tissue regions, further increasing the difficulty of achieving consistent global alignment. The selected FOVs were distributed across all three regions. For each region, CosMx-derived cell segmentation boundaries were overlaid on both the DAPI image and the aligned H&E image. The aligned cell boundaries closely matched histological cell structures, demonstrating accurate spatial correspondence at the cellular scale across both local regions and globally across disconnected tissue sections. Such cellular-scale alignment further enables more reliable interpretation of molecular measurements within histological kidney compartments, including glomerular and tubular regions, and may facilitate spatial localization of immune cell aggregates and other microenvironmental structures within complex renal tissue architecture.

To further assess robustness to incomplete spatial coverage, we performed a subsampling analysis by selecting different proportions of FOVs (80%, 60%, 40%, and 20%) for stitching and alignment (Methods, **Supplementary Fig. 5**). Despite substantial reductions in input data, PHARAOH produced highly consistent alignment results across sampling levels (Fig. 4c). Representative FOVs of interest showed minimal variation in spatial correspondence, indicating stable performance even when large portions of the FOVs are missing. Quantitative analysis (**Supplementary Table 3**) further supported this, with per-vertex displacements relative to full sampling (100%) remaining low across all subsampling levels (median shifts ranging from 4.13 to 6.63 pixels). Together, these results demonstrate that PHARAOH maintains robust alignment performance under substantially reduced spatial coverage, supporting its applicability to cost-efficient and spatially sparse experimental designs.

Overall, these results demonstrate that PHARAOH enables accurate, scalable, and user-friendly cross-modality registration for CosMx datasets, supporting reliable integration of high-resolution molecular measurements with histological context across large and spatially fragmented tissue sections.

### Spatial integration across adjacent tissue sections

Integration of ST datasets across adjacent tissue sections provides a practical and widely used strategy for approximating three-dimensional tissue organization and combining complementary spatial measurements across platforms. Because most ST technologies are performed on individual thin tissue sections and often rely on distinct assay-specific protocols, generating multi-platform measurements on the same physical section is typically infeasible. As a result, integration across adjacent sections enables reconstruction of spatial continuity across tissue depth and facilitates joint analysis of datasets generated using different experimental platforms.

Accurate spatial registration between adjacent sections is therefore essential for reliable integration. However, aligning adjacent tissue sections is substantially more challenging than registering images acquired from the same section. Although global tissue morphology is often preserved, local structural discrepancies frequently arise due to sectioning artifacts, tissue deformation, or partial tissue loss. Consequently, manual identification of corresponding landmarks across sections is time-consuming and unreliable. In addition, conventional global feature–based registration methods often struggle to identify stable correspondences because adjacent sections may contain substantial local structural differences.

**Fig. 5 |:**
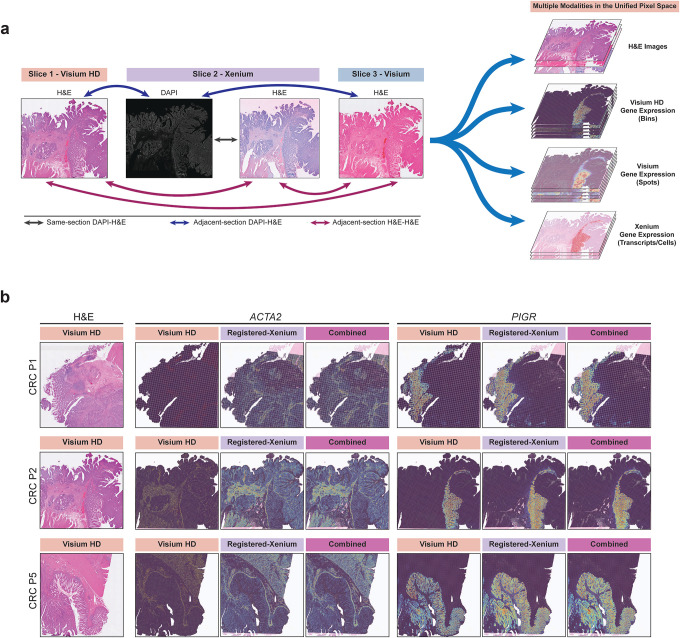
Cross-section integration enabled by PHARAOH for unified spatial transcriptomic analysis. **a,** Overview of PHARAOH for adjacent-section alignment under multiple modes, enabling mapping of ST data across sections into a unified pixel coordinate space. This framework supports integration of modalities such as Xenium, Visium, and Visium HD through histology-guided registration. **b,** Example of adjacent-section, cross-platform integration between Xenium and Visium HD. Xenium data are aligned to the Visium HD H&E image, and registered Xenium transcripts are aggregated into Visium HD 8 μm bins based on bin centers and spatial extent. UMI counts from Xenium are directly summed within each bin and integrated with Visium HD measurements at the bin level. For genes with weak or noisy expression in Visium HD (e.g., *ACTA2*), integration with Xenium enhances spatial patterns. For genes with partially concordant signals across platforms (e.g., *PIGR*), integration yields a more consistent spatial expression pattern.

To address these challenges, PHARAOH adopts a structure-guided tile matching strategy to identify locally corresponding tissue regions between adjacent slides (Methods). Instead of attempting nucleus-to-nucleus matching, which is generally infeasible across different sections, PHARAOH searches for image tiles exhibiting strong structural similarity across the two slides. These overlapping tiles represent regions where morphological structures are preserved between adjacent sections. The centroids of the matched tiles are subsequently used as spatial anchors to estimate the geometric transformation between slides, enabling robust cross-section alignment even when individual cellular correspondences are absent. This strategy is applicable across multiple alignment modes, including adjacent-section DAPI–H&E and H&E–H&E registration, as part of a unified framework for cross-section integration (Fig. 5a).

As an example, we applied this strategy to integrate adjacent ST datasets from a colorectal cancer sample (CRC-P2), combining Xenium DAPI images with Visium HD H&E sections (Fig. 5b, **Supplementary Fig. 6**). Despite differences in imaging modality and sectioning Z-plane, PHARAOH successfully identified structurally consistent regions across the two slides and established accurate spatial correspondence. The Xenium data were aligned to the Visium HD H&E image, and registered Xenium transcripts were aggregated into Visium HD 8 μm bins based on Visium HD bin centers and spatial extent. UMI counts from Xenium were directly summed within each bin and integrated with Visium HD measurements at the bin level, enabling joint spatial analysis within a unified coordinate system.

This integration enhances the interpretability of spatial gene expression patterns across platforms. For genes with weak or noisy expression in Visium HD (e.g., *ACTA2*), integration with Xenium reveals clearer spatial structure, whereas for genes with partially concordant signals across platforms (e.g., *PIGR*), integration produces more consistent and robust spatial patterns. Together, these results demonstrate that PHARAOH enables accurate cross-section alignment and effective integration of complementary spatial transcriptomics datasets.

### Generalization to multiplexed protein imaging modalities

Multiplexed protein imaging technologies generate high-dimensional spatial maps of protein expression by measuring dozens to hundreds of markers within intact tissue sections. These modalities are frequently integrated with histological staining to enable joint interpretation of molecular patterns and tissue morphology. Accurate cross-modality spatial alignment is therefore essential for linking molecular measurements with histopathological context and enabling integrative downstream analyses.

We evaluated PHARAOH using multiplexed protein imaging datasets from the RareCyte Orion^26^ platform across human colorectal adenocarcinoma samples. Orion is a single-pass imaging system in which all fluorescence channels are captured in one staining and imaging cycle, with nuclei visualized using Hoechst. The accompanying H&E image was provided with an existing alignment to the IF image, which we used as ground truth for benchmarking.

To quantitatively assess performance, we introduced controlled synthetic geometric distortions to the Orion IF images while keeping the H&E image fixed. Randomized transformations were applied and their inverses retained as ground-truth mappings (Methods). Results shown in Fig. 6a correspond to a representative sample, in which PHARAOH accurately recovered the imposed transformations, achieving a median restoration error of 3.29 pixels, compared with 115.64 pixels for VALIS. Consistent performance across additional samples is provided **in Supplementary Fig. 7**.

Representative regions of interest (ROIs) further illustrate these differences at cellular resolution (Fig. 6b). PHARAOH-restored images show precise spatial correspondence between Hoechst-stained nuclei and H&E morphology, with protein markers (e.g., PanCK, CD45, CD31, CD68, and PD-L1) remaining consistently aligned with underlying tissue structures. In contrast, VALIS exhibited clear spatial misalignment across multiple regions, with noticeable shifts in both nuclear and protein signal localization.

We next evaluated PHARAOH in adjacent-section settings using multiplexed immunofluorescence datasets generated by CODEX^27^ and CycIF^28^ (Fig. 6c, d). These cyclic imaging technologies involve repeated staining and imaging cycles, and in current practice, integration with histological staining often requires the use of adjacent tissue sections^7,29,30^. As a result, direct nucleus-to-nucleus correspondence is not expected due to sectioning-induced variability and local tissue deformation. Despite these challenges, PHARAOH successfully established accurate spatial alignment between fluorescence images and corresponding H&E sections. Across representative ROIs, major tissue structures are consistently aligned across modalities. While exact cellular correspondence is not preserved, protein marker distributions remain concordant with histological architecture, and exhibit biologically consistent spatial patterns across epithelial and stromal regions. Nuclear channels highlight expected discrepancies between adjacent sections, whereas protein channels remain well aligned, demonstrating robustness to cross-section variation.

Together, these results demonstrate that PHARAOH provides a generalizable registration framework that extends beyond ST to a broader class of spatial molecular imaging modalities. By leveraging nuclear structural signals and structure-guided regional matching, the framework enables reliable alignment between multiplexed fluorescence imaging and histological sections across both same-section and adjacent-section scenarios, supporting integrative analysis of tissue morphology and molecular measurements at high spatial resolution.

## Discussion

Accurate spatial registration between molecular imaging and histological sections is a prerequisite for integrative analysis in imaging-based ST. Here, we present PHARAOH, a hierarchical, nucleus-guided framework that enables robust and precise cross-modality alignment between fluorescence nuclear images and histological sections. By leveraging conserved nuclear geometry and integrating global initialization with local landmark refinement, PHARAOH achieves consistent nuclei-level correspondence across diverse datasets, tissue types, and imaging conditions.

A central strength of PHARAOH lies in its structure-driven design. Unlike modality-agnostic feature matching approaches, PHARAOH exploits the biological correspondence between nuclear fluorescence signals (e.g., DAPI or Hoechst) and hematoxylin staining. This provides a meaningful constraint for alignment and improves stability in challenging settings where global image features are weak or heterogeneous. Importantly, this structure-guided strategy enables less subjective selection of informative regions, thereby reducing potential user bias and improving the objectivity and reproducibility of the alignment process. Across Xenium datasets, this strategy yields tighter nucleus-level correspondence compared to generic frameworks, particularly in regions with complex morphology or low signal-to-noise ratio.

PHARAOH further benefits from a hierarchical multi-resolution architecture that balances global consistency with local precision. By combining coarse global alignment with tile-based refinement, the framework constrains local deformation while avoiding error propagation, enabling cellular-resolution alignment without computationally intensive global optimization or GPU acceleration. This design also improves robustness to staining variability and partial tissue degradation.

The framework demonstrates strong scalability and flexibility across experimental settings. In large, spatially fragmented CosMx data, PHARAOH achieves consistent global alignment where conventional methods are limited by memory or feature matching. It also extends to adjacent-section alignment via structure-guided regional matching, enabling cross-section integration even without one-to-one cellular correspondence. Beyond ST, PHARAOH generalizes to multiplexed protein imaging modalities, including Orion, CODEX, and CycIF, supporting consistent alignment across diverse imaging technologies.

Despite these strengths, several limitations remain. PHARAOH relies on a lightweight user-guided global initialization step, which leverages the ability of human operators to rapidly establish coarse structural correspondence across modalities. While this design improves robustness in challenging cases, it introduces a small degree of user interaction compared to fully automated approaches. In addition, the framework depends on sufficiently informative nuclear signals; in regions where nuclei are sparse, poorly stained, or structurally ambiguous, such as low-cellularity or matrix-rich tissues, alignment performance may be reduced. Addressing these scenarios may require incorporating complementary structural or multimodal features.

In summary, PHARAOH provides a robust, scalable, and generalizable framework for cross-modality spatial registration. By combining nuclear structural constraints with hierarchical alignment, it enables accurate mapping of molecular measurements into histological space, supporting integrative spatial analysis across platforms and experimental conditions.

## Methods

### Overview of the PHARAOH registration framework

PHARAOH performs cross-modality registration between nuclear-resolved fluorescence images and histological sections using a hierarchical multi-scale pipeline. The framework consists of three sequential stages: global initialization, nuclear landmark anchoring, and full-resolution transformation estimation.

### Image input and preprocessing

PHARAOH accepts two input images: a nuclear-resolved fluorescence image containing a nuclear channel (for example DAPI- or Hoechst-stained images) and a corresponding histological H&E image. For multi-channel fluorescence data, the nuclear channel can be specified by the user or, by default, the first channel is used. Input images can be provided in standard formats including OME-TIFF, TIFF, or JPEG. When multi-resolution OME-TIFF images are provided, PHARAOH automatically selects an intermediate pyramid level whose longest image dimension lies between 1,000 and 2,000 pixels. This intermediate-resolution representation enables efficient visualization and interactive global initialization while preserving sufficient structural detail.

To facilitate visual interpretation of nuclear structures during initialization, the nuclear fluorescence image is displayed using a lookup-table (LUT) transformation. By default, the Glasbey-inverted colormap is applied to enhance contrast between nuclei and background. Users can adjust the LUT intensity threshold to suppress low-intensity noise while preserving prominent nuclear structures, allowing clearer visualization of nuclear morphology.

Prior to registration, users are required to adjust the orientation of the nuclear image (including rotation and flipping) to approximately match the histological image. This step ensures consistent global orientation between modalities and facilitates subsequent alignment. These orientation adjustments are applied only to the visualization layer and do not alter the original image data. The processed mid-resolution nuclear image and the histological image are then used for global alignment in the subsequent stage.

### Global initialization

Following image preprocessing, PHARAOH performs an interactive global initialization step to establish coarse spatial correspondence between the nuclear fluorescence image and the histological image. During this step, the processed mid-resolution, LUT-transformed fluorescence image is used as the floating image and is overlaid onto the H&E image, which serves as the fixed reference.

PHARAOH provides two transformation modes for global initialization: affine and perspective. In affine mode, the floating image can be interactively translated and scaled to approximate the global spatial correspondence between the two modalities. Scaling can be applied either isotropically (uniform scaling along both axes) or anisotropically (independent scaling along the horizontal and vertical axes), allowing flexible adjustment of global tissue geometry. In perspective mode, the corner control points of the floating image can be adjusted to account for additional projective distortions that may arise during tissue processing or slide imaging. These transformations allow users to correct large-scale discrepancies in orientation, scale, and perspective between the two images.

Once the global tissue structures are visually aligned, the initialization parameters are saved as an initial geometric transformation. This transformation is represented as a 3 × 3 homography matrix, which is subsequently used to guide high-resolution tile extraction and local refinement in the following stages of the registration pipeline.

### Tile centroid sampling and tile extraction

Following global initialization, PHARAOH automatically identifies informative local regions for fine-scale alignment by sampling tile centroids from the nuclear image. This step is designed to select spatially distributed, nuclei-rich regions while maintaining broad coverage across the tissue.

To determine valid sampling regions, PHARAOH first constructs a tissue-support mask from the nuclear image at the sampling resolution. The nuclear image is converted to grayscale and smoothed with a Gaussian filter to generate a nuclear density map. Tissue regions are then identified by percentile-based thresholding of the density image, followed by morphological refinement and removal of small, connected components. To further exclude invalid areas introduced by image background or stitching boundaries, this density-derived tissue mask is intersected with a non-void mask derived from the original nuclear image. The resulting valid region is subsequently eroded by a radius determined from the tile size, ensuring that sampled centroids remain sufficiently far from boundaries so that the extracted tiles are fully contained within valid tissue regions.

Within this available region, PHARAOH samples tile centroids using a centroidal Voronoi tessellation (CVT)-based procedure. Candidate points are first initialized randomly under a minimum-distance constraint. The point set is then iteratively refined by assigning valid pixels to their nearest centroid and updating each centroid toward the center of its assigned Voronoi region. A soft separation constraint is applied during optimization to prevent excessive clustering of neighboring centroids. This procedure yields a set of spatially distributed centroids that provide balanced coverage of informative tissue regions.

Square tiles centered at the sampled centroids are then extracted from the nuclear image. Corresponding H&E regions are obtained by projecting the tile coordinates through the initial global transformation estimated in the previous stage. In practice, PHARAOH extracts rectified H&E tiles from the projected quadrilateral regions, enabling subsequent tile-level alignment between the two modalities. Tiles whose projected regions fall largely outside the H&E image are excluded before downstream processing.

### Nuclear mask generation for paired tiles

For each sampled tile pair, PHARAOH converts the nuclear and histological image patches into binary nuclear masks to facilitate morphology-driven local matching. Mask generation is performed independently for the fluorescence and H&E tiles using fast, straightforward, and foundation model–free procedures. This design avoids reliance on complex pretrained models and enables efficient, scalable processing while preserving essential nuclear structural information.

For nuclear fluorescence tiles, PHARAOH uses the intensity-normalized grayscale (8-bit) nuclear tile extracted from the fluorescence image. A binary foreground mask is generated using Otsu thresholding with an optional intensity offset, allowing threshold adjustment based on pilot preview parameters. The resulting mask is refined by hole filling and removal of small, connected components.

For H&E tiles, PHARAOH applies a simple intensity-based dark-nuclei segmentation procedure to identify hematoxylin-enriched nuclear regions. Specifically, the H&E tile is processed using a threshold-based routine that detects strongly stained nuclear regions while suppressing lighter background tissue structures. This design avoids dependence on foundation models or other large pretrained segmentation frameworks and instead provides a lightweight and efficient representation of nuclear morphology for downstream matching.

Both fluorescence and histological tiles are upsampled prior to mask generation to improve spatial resolution and robustness in downstream local alignment. The resulting binary masks provide simplified, morphology-driven representations of nuclear structure while reducing modality-specific appearance differences between fluorescence and histological images.

### Tile-level alignment and anchor identification

PHARAOH next performs local alignment for each paired fluorescence and histological mask tile. Because the global initialization step already establishes approximate cross-modality correspondence, local refinement is restricted to isotropic scaling and translation. For each tile pair, the histological mask is first rescaled and padded to match the fluorescence tile canvas. A two-stage search procedure is then applied. In the first stage, a coarse search is performed on downsampled masks across a predefined range of isotropic scales and translations to identify an approximate local transformation that maximizes an overlap-based matching score between the two masks. In the second stage, this solution is refined at full resolution within a narrower search window using the same scoring criterion to obtain the final tile-level alignment.

In practice, the matching score is defined as the Dice coefficient between the fluorescence mask and the transformed histological mask. For a fluorescence tile mask *M*_*F*_ and transformed histological tile mask *M*_*H*_, the tile-level matching score is defined as

$$\text{Dice}\left(M_{F},M_{H}\right)=\frac{2\times \text{area}\left(M_{F}\cap M_{H}\right)}{\text{area}\left(M_{F}\right)+\text{area}\left(M_{H}\right)}$$

Following local alignment, PHARAOH identifies candidate anchor nuclei from isolated connected components within each aligned tile pair. Connected components are extracted independently from the fluorescence and histological masks and filtered according to minimum size, spatial isolation, and centrality within the tile. Candidate fluorescence and histological nuclei are then paired based on spatial overlap after alignment.

To quantify pairwise correspondence at the component level, we adopt the same Dice formulation. For a candidate fluorescence component *F*_*i*_ and histological component *H*_*j*_, we defined directional coverages as

$${\text{score}}_{ij}=\frac{2\times \text{area}\left(F_{i}\cap H_{j}\right)}{\text{area}\left(F_{i}\right)+\text{area}\left(H_{j}\right)}$$

This unified Dice-based formulation is applied consistently at both the tile and component levels. Importantly, it naturally penalizes asymmetric overlap (e.g., a small component fully contained within a much larger one), ensuring that high scores are assigned only to pairs with well-matched size and spatial extent. Candidate pairs are further required to satisfy minimum overlap, size, and isolation constraints.

Tiles containing sufficient numbers of reliable nucleus pairs, as defined by user-specified minimum pair count and overlap-based quality criteria, are retained as high-confidence anchor tiles. For these tiles, the centroids of the matched nuclei are used as local anchor correspondences. If too few tiles satisfy these criteria, PHARAOH falls back to tiles with sufficiently strong overall mask agreement and uses tile centers as surrogate anchors. For all retained anchors, coordinates are mapped from the aligned tile coordinate system back to the original fluorescence and histological image spaces. The resulting set of matched nuclear centroids or tile-center correspondences is then used in the next stage to estimate the final cross-modality transformation.

### Nucleus patch extraction and optional centroid refinement

Following anchor identification, PHARAOH extracts local nucleus-centered patches around each retained anchor pair for visualization and quality assessment. Anchor coordinates are mapped from tile space back to the original fluorescence and histological image coordinate systems, accounting for pyramid levels and tile offsets. Local patches are then cropped from the fluorescence and H&E images around the mapped centroids.

For each extracted patch, PHARAOH saves both the raw image patch and a centroid-marked overlay for visualization. The extracted patch gallery enables users to inspect candidate anchor pairs, optionally adjust centroid locations, and exclude unreliable anchor pairs prior to final transformation estimation.

### Full-resolution transformation and coordinate propagation

Using the reviewed anchor points, PHARAOH then estimates the final full-resolution transformation between the nuclear image and the histological reference frame. Depending on the selected transformation mode, the final mapping can be represented as an affine transform, a homography, or a thin-plate spline (TPS) model.

Once the final transformation is estimated, PHARAOH enables a range of downstream operations based on the aligned coordinate systems. These include optional warping of the nuclear fluorescence image into the histological reference frame to generate fluorescence–histology overlays for visual assessment, as well as transformation of spatial features across multiple levels, including cell centroids, nucleus boundaries, cell boundaries, and transcript coordinates.

The propagated coordinates are retained in the full-resolution histological coordinate system and can be used for downstream visualization and quantitative analysis. PHARAOH further provides an interactive visualization module that displays registered fluorescence overlays, aligned anchor points, and transformed spatial features directly on the histological image. These outputs provide a final quality-control step for assessing registration accuracy and enable direct interpretation of molecular and cellular measurements within tissue morphology.

### Extension to histology–histology registration

PHARAOH can be extended to histology–histology registration by replacing the fluorescence-derived nuclear reference with hematoxylin-derived structural cues. In this setting, the floating image is not represented by a fluorescence nuclear channel, but instead by a hematoxylin-enhanced image together with a threshold-derived structural mask that highlights hematoxylin-positive regions.

Compared with the DAPI–H&E pipeline, the principal difference lies in the definition of the available sampling region. In the fluorescence-based setting, sampling regions are determined from nuclei-enriched areas identified from the nuclear image and tissue-support masks. In the histology–histology setting, by contrast, the available sampling region is defined directly from the thresholded hematoxylin-positive mask of the floating histology image, followed by morphological refinement and inward boundary filtering using a distance transform.

This adaptation preserves the overall hierarchical registration framework of PHARAOH while replacing fluorescence-derived nuclear support with hematoxylin-derived structural support, thereby enabling registration between adjacent histological sections and other histology-only integration scenarios.

### VALIS benchmarking and configuration

For comparison with VALIS, DAPI and H&E images from each dataset were provided as paired inputs to VALIS, with the H&E image specified as the registration reference. VALIS was run under a standardized computational setting across all datasets without dataset-specific parameter tuning. Parameter settings were kept consistent across datasets to maintain a standardized and practically deployable benchmarking setting rather than performing extensive dataset-specific optimization. To ensure compatibility with the VALIS input pipeline, microscopy images that could not be directly processed by VALIS (e.g., certain OME-TIFF files) were converted into LZW-compressed BigTIFF files prior to registration. Registration was performed using VALIS with max_image_dim_px = 1024, max_processed_image_dim_px = 1024, and max_non_rigid_registration_dim_px = 1024. The resulting transformation matrix was exported and used for downstream coordinate transfer and cross-modality spatial mapping.

All VALIS analyses were performed on a standard MacBook Pro under the same computational setting used for PHARAOH benchmarking. Runtime was typically approximately 5–10 min per Xenium dataset, including input conversion and matrix export. Failed cases were re-run to assess reproducibility and showed consistent behavior across runs. Failure modes included insufficient or unstable feature correspondences for reliable transformation estimation, severely distorted alignments, and memory exhaustion during processing.

### Evaluation metrics and baseline comparison

We evaluated the alignment performance of PHARAOH using both quantitative and qualitative criteria. Quantitative evaluation focused on the spatial consistency between transformed Xenium-derived features and nuclear regions segmented from the H&E image using HoverNet^31^. Specifically, we used two quantitative metrics.

First, for each transformed Xenium-measured nucleus polygon *i*, we computed a nucleus coverage score *s*_*i*_ defined as

$$s_{i}=\frac{\text{area}\left(\text{Xenium}{\;\text{polygon}}_{\text{i}}\cap H\text{overNet mask}\right)}{\text{area}\left(\text{Xenium}{\;\text{polygon}}_{\text{i}}\right)}$$

This metric measures the fraction of the transformed Xenium nucleus polygon that overlaps the HoverNet-segmented H&E nuclear mask.

Second, for each transformed transcript *j*, we computed the Euclidean distance to the nearest HoverNet-segmented nuclear region in H&E space, defined as

$$d_{j}={min}_{x\in N_{\left\{\text{HoverNet}\right\}}}{\left\|t_{j}-x\right\|}_{2}$$

where *t*_*j*_denotes the transformed coordinate of transcript *j*, and *N*_{*HoverNet*}_ represents the set of pixels belonging to the HoverNet-segmented nuclear mask.

For nucleus coverage score comparisons between PHARAOH and VALIS, we summarized paired regional differences using three complementary statistics: the median paired difference in coverage score, the fraction of regions in which PHARAOH achieved higher coverage scores than VALIS, and the matched-pairs rank biserial correlation *r*_*rb*_. The matched-pairs rank biserial correlation was calculated as

$$r_{rd}=\frac{1}{n}\sum_{i=1}^{n}\text{sign}\left(s_{i}^{\text{PHARAOH}}-s_{i}^{\text{VALIS}}\right)$$

where $s_{i}^{\text{PHARAOH}}$ and $s_{i}^{\text{VALIS}}$ denote the paired coverage scores for region *i*.

For transcript-to-nearest-nucleus distance analysis, we summarized distribution differences using three complementary measures. First, median transcript-to-nucleus distances were compared between methods to quantify the typical magnitude of spatial displacement. Second, overall distributional differences were quantified using the two-sample Kolmogorov–Smirnov (KS) statistic, defined as the maximum absolute difference between the empirical cumulative distribution functions of the two methods. Third, we computed the common-language effect size (CLES), defined as the probability that a randomly sampled PHARAOH transcript-to-nucleus distance was smaller than a randomly sampled VALIS distance. Due to the large number of transcript-level observations, CLES was estimated using a random subsample of 100,000 observations, with ties weighted at 0.5.

We compared PHARAOH against VALIS, a widely used framework for whole-slide image registration. Evaluation was performed across multiple datasets and regions of interest (ROIs), with identical inputs, transformed coordinates, and H&E-derived nuclear masks used for both methods to ensure a fair comparison.

In addition to quantitative metrics, visual assessment was conducted by overlaying transformed Xenium nucleus boundaries onto hematoxylin-stained nuclei in H&E images, providing an intuitive evaluation of alignment quality across different tissue contexts.

### Usability Evaluation and Runtime Characterization

To evaluate usability and user-dependent variability, we conducted a user study involving three independent test users with no prior experience using PHARAOH, in addition to the author. Each participant performed the interactive initialization procedure on a set of 16 Xenium datasets across diverse computing environments and operating systems. For comparison, an independent user performed manual alignment on a subset of three datasets using Xenium Explorer as a baseline for fully manual registration. The manual alignment procedure and timing definitions are described in **Supplementary Methods**.

Timing information was recorded throughout the workflow to quantify both system processing time and user interaction time. To ensure a fair and standardized assessment, we defined a runtime metric that excludes prolonged periods of user inactivity (e.g., interruptions unrelated to the workflow). Specifically, intervals between consecutive user actions exceeding 10 seconds were truncated and not counted toward user interaction time. Under this definition, total runtime is decomposed into system processing time and user interaction time, with the latter further including manual alignment and other active operations.

For the user study, a fixed configuration of 120 sampled tiles (as specified in parameters.json) was used across all datasets to ensure consistency of evaluation. Detailed timing results for all users and datasets are provided in **Supplementary Table 2**, and dataset descriptions are provided in **Supplementary Table 1**. A complete description of the timing definitions, user study protocol, and implementation details is provided in **Supplementary Methods**.

### Subsampling analysis for incomplete spatial coverage

To evaluate the robustness of PHARAOH under reduced spatial coverage, we performed a field-of-view (FOV) subsampling analysis using a NanoString CosMx dataset containing 483 total FOVs. Because the initial manual alignment step in PHARAOH relies on recognizable global tissue geometry, a small subset of structurally informative FOVs located along distinctive tissue contours or anatomical landmarks was always retained across all subsampling settings. This design reflects practical experimental usage, where FOV placement is user-defined and users would typically preserve visually informative regions necessary for reliable global initialization. The specific FOVs retained and subsampled for each coverage level are shown in **Supplementary Fig. 5**.

After preserving these structure-informative FOVs, the remaining FOVs were randomly subsampled to generate datasets corresponding to 80%, 60%, 40%, and 20% spatial coverage relative to the full dataset (100%). For each sampling level, the selected FOVs were stitched to generate the corresponding morphology image, followed by the standard PHARAOH registration pipeline. Alignment consistency was assessed by comparing transformed vertex coordinates from each subsampled condition against the full-coverage registration result (100%). Per-vertex Euclidean displacement statistics were calculated across all registered vertices for each sampling proportion, including mean, median, and maximum pixel shifts (**Supplementary Table 3**).

### Synthetic distortion and evaluation framework for Orion IF registration

#### Synthetic distortion.

A randomized geometric transformation was applied to the Orion IF image to simulate misalignment, while the corresponding H&E image was kept fixed. The same transformation was applied consistently across all IF channels. The transformation included translation, rotation, scaling, shear, and mild perspective perturbations, with parameters sampled within predefined ranges. The inverse transformation was retained as the ground-truth for evaluation. The transformation was applied directly to each level of the pyramidal OME-TIFF image. For large images, tiled warping was used to ensure computational efficiency and to avoid size limitations. Border regions introduced during warping were filled with zero intensity.

#### Sampling and evaluation.

Registration accuracy was evaluated using grid-based sampling in the distorted IF image space. Points were sampled at regular spatial intervals, and only those mapping to valid coordinates in the reference space were retained. For each sampled point, the restored coordinate obtained from a given registration method was compared to the ground-truth coordinate defined by the inverse transformation. The restoration error was defined as the Euclidean distance between these coordinates. Performance was summarized using the distribution of restoration errors across all sampled points, including median, mean, and percentile-based metrics.

## Supplementary Material

Supplementary Files

This is a list of supplementary files associated with this preprint. Click to download.

- supfigure4.pdf
- supfigure6.pdf
- SupplementaryAll.docx
- supfigure7.pdf
- supfigure1.pdf
- supfigure2.pdf
- supfigure3.pdf
- supfigure5.pdf

## Figures and Tables

**Fig. 6 |:** PHARAOH enables accurate mIF–H&E registration across in-sample and adjacent-section settings. **a**, In-sample registration of Orion mIF data to H&E using the Hoechst nuclear channel. Six representative protein channels spanning epithelial, immune, and stromal markers are shown, each with three distinct regions of interest (ROIs) indicated on the H&E image (left). For each channel, corresponding H&E patches and overlays of aligned fluorescence signals are shown (right), demonstrating accurate spatial correspondence across diverse markers. **b,** Adjacent-section registration of CODEX data to H&E using the DAPI channel. Left, schematic of cross-section alignment. Right, H&E and aligned overlays for five representative protein channels covering diverse tissue compartments across the same ROIs. Despite expected cell-level variation between adjacent sections, all channels exhibit consistent and biologically plausible alignment, while the nuclear channel highlights expected discrepancies due to section mismatch. **c,** Adjacent-section registration of CycIF data to H&E using the first DNA channel. Left, schematic of alignment. Right, H&E and aligned overlays for five representative protein channels covering diverse tissue compartments across the same ROIs. As in (**b**), nuclear signals reveal expected cross-section differences, whereas protein markers remain well aligned, demonstrating robustness of PHARAOH to adjacent-section variation.

## Data Availability

We analyzed the following datasets in this study: (1) 10x Genomics human colorectal cancer datasets, including Visium HD datasets (CRC-P1, CRC-P2, and CRC-P5) and the corresponding Xenium datasets from the same samples (https://www.10xgenomics.com/platforms/visium/product-family/dataset-human-crc). For CRC-P2, an additional Visium dataset was included to enable cross-platform integration. (2) 10x Genomics human Xenium datasets across multiple tissues, including liver cancer (https://www.10xgenomics.com/datasets/human-liver-data-xenium-human-multi-tissue-and-cancer-panel-1-standard), lung cancer (10x dataset: https://www.10xgenomics.com/datasets/visium-hd-cytassist-gene-expression-human-lung-cancer-post-xenium-expt), and additional lung adenocarcinoma samples (LUAD S1-S5; https://zenodo.org/records/18165537). (3) 10x Genomics human breast cancer Xenium datasets, including standard breast cancer data (https://www.10xgenomics.com/products/xenium-in-situ/preview-dataset-human-breast) and Xenium Prime 5K breast cancer data (https://www.10xgenomics.com/datasets/xenium-prime-ffpe-human-breast-cancer). (4) 10x Genomics human artery Xenium datasets (P4D and P4H; https://zenodo.org/records/18165537). (5) 10x Genomics mouse Xenium datasets, including mouse colon (https://www.10xgenomics.com/datasets/fresh-frozen-mouse-colon-with-xenium-multimodal-cell-segmentation-1-standard) and mouse brain (S1) (https://www.10xgenomics.com/datasets/fresh-frozen-mouse-brain-for-xenium-explorer-demo-1-standard). (6) NanoString CosMx human kidney cancer dataset (HK2844–3039) previously reported in Dumoulin et al.^25^ (https://zenodo.org/records/17228449). (7) RareCyte Orion multiplexed protein imaging datasets from human colorectal cancer samples, including descending colon adenocarcinoma (HTA7_919_6) and rectal/rectosigmoid adenocarcinoma samples (HTA7_920_9 and HTA7_934_9), available from the Human Tumor Atlas Network (https://humantumoratlas.org/publications/hta7_2022_cell_jia-ren-lin?tab=overview). (8) CODEX dataset from human colon adenocarcinoma (https://spatch.pku-genomics.org/#/dataset/xenium). (9) CycIF dataset from human cecum mucinous adenocarcinoma (HTA13_1_106) (https://humantumoratlas.org/publications/hta7_2022_cell_jia-ren-lin?tab=overview). Details of all datasets analyzed in this study are provided in **Supplementary Table 1**.

## Code Availability

PHARAOH was implemented in Python and is available on GitHub at https://github.com/yaosicong1999/PHARAOH. All alignment outputs for the analyzed datasets, including user-initialized coarse transformations and final estimated transformation matrices, are provided in the repository.
